# Serum Vitamin D Level as a Risk Factor and Prognostic Marker for Acute Ischemic Stroke: A Case-Control Study at a Tertiary Care Centre in Northern India

**DOI:** 10.7759/cureus.46117

**Published:** 2023-09-28

**Authors:** Kislaya Kamal, Jay Tewari, Vipin Bharti, Deepak Sharma, Isha Atam, Virendra Atam, Anadika Rana, Shubhajeet Roy

**Affiliations:** 1 Internal Medicine, King George's Medical University, Lucknow, IND; 2 Medicine, King George’s Medical University, Lucknow, IND; 3 Internal Medicine, King George’s Medical University, Lucknow, IND; 4 Medicine, King George's Medical University, Lucknow, IND

**Keywords:** stroke, prognostic marker in stroke, risk factor, acute ischemic stroke, vitamin d

## Abstract

Introduction

Stroke is a predominant cause of death worldwide. Major risk factors for stroke in any age group are diabetes, hypertension, heart disease, smoking, and long-term alcohol abuse. It is of utmost importance to identify the risk factors for stroke to prevent recurrence. Vitamin D deficiency is identified as a risk factor for stroke. Therefore, we attempted to look for a correlation between vitamin D levels and acute ischemic stroke.

Methods

This observational case-control study was conducted with 150 patients (75 cases and 75 controls). On the day of admission, the National Institutes of Health Stroke Scale (NIHSS) score was calculated, and vitamin D levels were measured for each patient. The functional outcome was determined by the modified Rankin scale (mRS).

Results

The most common risk factors identified in this study were hypertension (61.3%), diabetes mellitus (41.3%), and smoking (37.3%). Out of 75 patients enrolled in the study, 49.4% had significant vitamin D deficiency, and 30.6% had insufficient vitamin D levels. Our study showed a significant correlation between vitamin D sufficiency in the body and the incidence of stroke (x^2^=3.888 and p=0.048). A significant correlation (p=0.03) was found between the NIHSS score and vitamin D levels in patients with acute ischemic stroke.

Conclusion

In this observational case-control study, we concluded that the increasing severity of vitamin D deficiency was associated with more deaths and poor outcomes.

## Introduction

The World Health Organization defines stroke as “rapidly developing clinical signs of focal (or global) disturbance of cerebral function, with symptoms lasting 24 hours or longer or leading to death, with no apparent cause other than of vascular origin” [1]. Stroke is the world’s fifth leading cause of death, as per the CDC data for 2023 [2].

Stroke is a complex condition, and it is necessary to monitor its various associated signals [3]. The most common stroke symptom is sudden weakness or numbness of the face, arm, or leg, usually on one side of the body, which occurs in 90% of strokes. A stroke can affect people of any age but is most common in the elderly (those over 65). The mortality rate of stroke in the acute phase can reach 20%, and it remains higher in stroke patients for several years after the acute event than in the general population. Stroke patient recovery can take months or years at best, putting a strain on the healthcare system in developed countries around the world [4]. While subarachnoid and intracranial hemorrhage cause a higher proportion of strokes in young adults (40% to 55%) than in the general stroke population (15% to 20%), cerebral infarction remains the most common [3].

Ischemic stroke is mostly preventable, and the risk factors are similar in developed and developing countries [5]. Diabetes, hypertension, heart disease, current smoking, and long-term heavy alcohol consumption are all major risk factors for stroke in both young adults and the elderly [6,7]. It is critical to identify the underlying causes of the stroke to prevent recurrences. It is well known that there are two main types of brain stroke: hemorrhagic stroke (brain hemorrhage), which occurs due to leakage or rupture of the blood vessel causing bleeding in the brain; and ischemic stroke, which is caused by a blockage in the arterial blood supply to the brain. Ischemic stroke and hemorrhagic stroke account for approximately 87% and 13% of all brain strokes, respectively [8,9]. Therefore, it is important to identify the causative factors in ischemic stroke patients to prevent recurrences. However, some local risk factors, such as acute injury to the brain, extreme cases of meningitis, and encephalitis, can cause brain damage, stroke, or even death [7].

Vitamin D deficiency is a new but very important systemic risk factor for brain stroke (cerebrovascular accident) and has now come to the fore, as shown by some of the literature in the last few years [10,11]. This is because vitamin D deficiency plays an important role in the pathogenesis of stroke through endothelial dysfunction of the carotid artery that supplies blood to the brain [12]. Following the discovery of the expression of vitamin D receptors and 1-α-hydroxylase in the endothelium of blood vessels, several biological mechanisms that link vitamin D to stroke and its risk factors have been identified [10].

As the aforementioned literature suggests, vitamin D plays a major role in the prevention of brain stroke, and among all types of brain stroke, ischemic stroke has the highest percentage of prevalence. Therefore, a better and more advanced exploration of the role of vitamin D as a risk factor and prognostic marker in patients with acute ischemic stroke becomes more important. Vitamin D helps in protection and recovery from ischemic stroke in several ways, such as by reducing hypertension [13], by negative regulation of the renin-angiotensin system [14], by increasing intimal media thickness and stabilizing carotid plaques in cardioembolic stroke [15,16], and by increasing antithrombotic and neuroprotective factors [17,18]. Vitamin D acts mainly through its role in maintaining gene transcription to prevent cerebrovascular disease and its risk factors [19]. Prabhakar et al. found that the increased risk of stroke in vitamin D-deficient individuals was associated with the genetic variation in the vitamin D receptor gene [20].

As we know, acute ischemic stroke is the opportune time to check the impact of 25-hydroxy vitamin D (25(OH)D) levels on it. Therefore, this study was designed to assess the vitamin D levels in patients with acute ischemic stroke and to find any significant correlation, if present. We measured the serum 25(OH)D levels in patients with acute ischemic stroke and compared their levels with age and sex-matched controls. We also analyzed the severity of stroke based on the National Institutes of Health Stroke Scale (NIHSS) and examined the functional outcome of patients at the time of discharge based on the modified Rankin scale (mRS).

Since the level of vitamin D is directly measurable, its deficiency can also be treated. Several trials are being conducted around the world to assess its association with and preventive role in ischemic stroke. Despite the increasing proportion of ischemic strokes in Indians, only limited data on the relationship between vitamin D and ischemic stroke is available. India is a diverse country with many regional differences in race, culture, disease spectrum, etc. This study will serve as a representation of the northern Indian state of Uttar Pradesh, where the data regarding this topic is especially lacking.

## Materials and methods

A prospective observational case-control study was conducted at the Department of Internal Medicine, King George's Medical University, Lucknow, India. This study was approved by the Institutional Ethics Committee (approval no. ECR/262/Inst/UP/2013/RR-19). A total of 150 consenting patients (75 cases of acute ischemic stroke and 75 controls) were enrolled in the study between March 2021 and August 2022. The study included all patients above the age of 12 who fulfilled the WHO definition of stroke and were documented by radiological investigations (CT and/or MRI) as cases, and patients without stroke who attended the medicine outpatient department were enrolled as controls (risk factors, age, and sex-matched). Patients with hemorrhagic stroke, transient ischemic attacks, and patients whose clinical and radiological findings were not consistent with stroke, had a head injury, intracranial space-occupying lesion, chronic kidney disease, or were on drugs affecting vitamin D metabolism (anti-epileptics, steroids) or on calcium and vitamin D supplements were excluded from the study.

All the participants were evaluated with a detailed history, clinical examination, and severity assessment of stroke per NIHSS score at the time of admission. The detailed clinical history included all the symptoms of stroke, with an emphasis on the risk factors attributable to ischemic strokes like smoking, alcohol consumption, diabetes, hypertension, dyslipidemia, coronary artery disease, and rheumatic heart disease. A detailed clinical examination was done to identify the neurological deficits. The NIHSS score at the time of admission was calculated, and the severity was graded as minor stroke (NIHSS=1-4), moderate stroke (NIHSS=5-15), moderate to severe stroke (NIHSS=16-20), and severe stroke (NIHSS=21-42). All the participants were subjected to the relevant investigations depending upon the suspected etiology of stroke, viz., complete blood count with differential and platelet count, random blood glucose, serum electrolytes (sodium, potassium, and calcium), kidney function tests, liver function tests, glycated hemoglobin, lipid profile, electrocardiogram, coagulation profile, etc.

The levels of 25(OH)D3 were assessed in both cases and controls by chemiluminescent microparticle immunoassay in the chemical pathology lab of our hospital. The levels of vitamin D were graded according to lab values as normal (30.1-100 ng/ml), insufficiency (10.1-30 ng/ml), deficiency (<10 ng/ml), and intoxication (>100 ng/ml).

The cases were managed accordingly during their hospital stay, and the outcome at discharge was noted. The functional outcome of the patients was determined by the mRS (Table 1). An mRS score of ≤ 2 was considered a good outcome, and a score >2 was considered a poor outcome.

**Table 1:** The modified Rankin scale

Score	Description
0	No symptoms
1	No significant disability. Able to carry out all usual activities, despite some symptoms.
2	Slight disability. Able to look after own affairs without assistance, but unable to carry out all previous activities.
3	Moderate disability. Requires some help, but can walk unassisted.
4	Moderately severe disability. Unable to attend to own bodily needs without assistance, and unable to walk unassisted.
5	Severe disability. Requires constant nursing care and attention, bedridden, incontinent.
6	Expired

The data collected regarding all the selected cases and controls was recorded and analyzed with SPSS Statistics version 19.0 (IBM Corp., Armonk, NY, USA). The measured data were presented as the mean ± standard error of the mean. The t-test and chi-square test were used to analyze the data gathered. A value of p <0.05 was considered statistically significant.

## Results

We included 75 cases of acute ischemic stroke in this study (45 males (60%)) (Table 2). The most common etiology identified was hypertension (46; 61.3%), followed by diabetes mellitus (31; 41.3%), and smoking (28; 37.3%) (Table 3). Out of 75 cases, 41 patients had a severe stroke, and 30 patients had a mild stroke per the NIHSS severity score (Table 4). 

**Table 2:** Age and gender of cases and controls enrolled in the study

Characteristics		No. of Cases (percentage)		No. of Controls (percentage)	
Age	Young (18 to 39 years)	13 (17.33%)		16 (21.33%)	
	Middle age (40 to 59 years)	19 (25.33%)		15 (20%)	
	Old age (above 60 years)	43 (57.33%)		44 (58.66%)	
Sex	Male	45 (60%)		41 (54.66%)	
	Female	30 (40%)		34 (45.33%)	

**Table 3:** Risk factors identified in cases and controls enrolled in the study

Risk Factor	No. of Cases	Percentage of Cases	No. of Controls	Percentage of Controls
Smoking	28	37.3%	22	29.3%
Chronic alcoholics	22	29.3%	19	25.3%
Diabetes mellitus	31	41.3%	29	38.6%
Hypertension	46	61.3%	40	53.3%
Hyperlipidemia	24	32.0%	21	28%

**Table 4:** Severity of stroke based on NIHSS severity score NIHSS: National Institutes of Health Stroke Scale

NIHSS Category (Score) of Enrolled Subjects	No. of Subjects	Percentage
Minor stroke (1-4)	1	1.33%
Mild stroke (5-15)	30	41.33%
Moderate to severe stroke (16-20)	3	4%
Severe stroke (25-42)	41	54.67%

The mean vitamin D level in the control arm was 28.57±42.84 ng/ml, and cases had a mean vitamin D level of 19.79 ± 10.79 ng/ml. Out of 75 cases, a deficient level of the vitamin was found in 44 patients, and 23 patients had vitamin D levels in the insufficient range (Table 5).

**Table 5:** Serum vitamin D assay in cases and controls enrolled in the study

Vitamin D level	No. of Cases (Percentage)		No. of Controls (Percentage)	
Highly deficient (˂10 ng/mL)	7 (9.3%)		5 (6.6%)	
Deficient (10 to 20 ng/mL)	37 (49.4%)		23 (30.6%)	
Insufficient (Between 20 to 30 ng/mL)	23 (30.6%)		30 (40%)	
Normal (30 to 50 ng/mL)	7 (9.3%)		15 (20%)	
High (˃50 ng/mL)	1 (1.4%)		2 (2.6%)	
Total	75 (100%)		75 (100%)	

A correlation between the incidence of stroke and the sufficiency of vitamin D in the body was analyzed using the chi-square test, and we found a significant association between the two (p=0.048) (Table 6).

**Table 6:** Correlation between the incidence of stroke and vitamin D sufficiency in patients enrolled in cases and controls X^2^: Chi-square, p: p-value

Correlation of Vitamin D Levels	Cases	Controls
Vitamin D ≥30 ng/dL	8	17
Vitamin D ≤30 ng/dL	67	58
X^2^	3.888	
p	0.048	

In this study, we calculated the NIHSS score at the time of admission, and it was found that deficiency of vitamin D had a direct correlation (p=0.03) with the NIHSS score (Table 7).

**Table 7:** Correlation between vitamin D category and NIHSS score NIHSS: National Institutes of Health Stroke Scale

NIHSS Score	No. of Subjects in Each Vitamin D Category					Pearson Coefficient	p-value
	Highly deficient (˂10 ng/mL)	Deficient (10 to 20 ng/mL)	Insufficient (Between 20 to 30 ng/mL)	Normal (30 to 50 ng/mL)	High (˃50 ng/mL)		
1 – 4	0	0	1	0	0	-0.2459	0.0335
5 – 15	2	14	10	3	1		
16 – 20	0	2	1	0	0		
21 – 42	5	21	11	4	0		
	7	37	23	7	1		

The mRS score was calculated for each patient at the time of discharge. Patients with vitamin D deficiency had an mRS score of >2 with a p-value <0.0001, which is statistically significant (Table 8).

**Table 8:** Correlation between serum vitamin D level and mRS at the time of discharge mRS:  Modified Rankin scale

The mRS Score of Subjects at Discharge	No. of Stroke Subjects According to Serum Vitamin D Level					Spearman Coefficient	p-value
	Highly deficient (˂10 ng/mL)	Deficient (10 to 20 ng/mL)	Insufficient (between 20 to 30 ng/mL)	Normal (30 to 50 ng/mL)	High (˃50 ng/mL)		
1	0	0	1	0	0	-0.6427	< 0.0001
2	0	0	5	1	1		
3	0	4	5	2	0		
4	1	14	4	1	0		
5	4	10	3	0	0		
6 (Expired)	2	9	5	3	0		

## Discussion

Ischemic stroke is a rapidly progressing neurological disease that, in addition to being a medical emergency, contributes to an ever-increasing global mortality and morbidity burden. Biomarkers are objective indicators that are used to evaluate normal or pathological processes and treatment responses and to predict or assess prognosis. Numerous biomarkers related to acute ischemic stroke have been identified and studied, but none of the biomarkers are currently available for the prognosis of acute ischemic stroke. So, in this study, we measured 25(OH)D levels and assessed their relationship with stroke severity at the time of admission per the NIHSS score, while the functional outcome of subjects at discharge was evaluated according to the mRS.

In our study, known risk factors including diabetes mellitus, hypertension, smoking, and alcohol were found in the proportion of 41.3%, 61.3%, 37.3%, and 29.3% of subjects, respectively. The mean NIHSS score was 20.73 ± 9.444, and it was also revealed that deficient vitamin D levels were associated with higher NIHSS scores (p-value of 0.0335). From the 75 cases enrolled in the study, 56 survived and 19 expired (the case fatality rate was 25.3%) within three months of follow-up. This is in concordance with a study by Pandian and Sudhan, who reported a more than 20% mortality rate in their study [20]. The slightly high mortality and the smaller number of minor strokes can be due to our hospital being a tertiary care government hospital, with most of the patients being referred from other hospitals.

In the current study, mean serum vitamin D levels were found to be deficient in stroke patients (19.79 ± 10.79 ng/ml) as compared to controls (28.57 ± 42.84 ng/ml). This is in correlation to the study done by Selim et al. (2019) [21], who reported that a serum vitamin D level of ≤17 ng/ml was shown to predict stroke outcomes. The link between vitamin D and hospital stay duration was not found to be significant (Pearson coefficient =-0.1784; p= 0.1883) since the majority of patients were discharged within 14 days of admission, regardless of vitamin D level.

The prognostic value of vitamin D was tested in association with the mRS score at the time of discharge. It was discovered that the vitamin D deficient category (10 to 20 ng/ml) had the most stroke participants with a mRS score of >2. We also observed that the proportion of deaths was higher in patients with low serum vitamin D levels, implying that the severity of vitamin D deficiency was associated with more deaths and a poor mRS score at the time of discharge (p <0.0001 ). However, more studies with larger sample sizes are required to arrive at conclusions. The NIHSS score at admission and the mRS score at discharge were shown to be directly proportional to each other, with a highly significant link (Spearman correlation coefficient = 0.7799; p=0.0001). As a result, the NIHSS score upon admission can be a useful tool for predicting functional prognosis. This is in contrast to the study of Peters et al. (2015) [22], which revealed no link between the NIHSS score and the MRS score. Menon et al. (2016) [23] likewise found no link between functional outcome and the NIHSS score. 

Vitamin D receptors are known to be expressed in most human cells, including vascular smooth muscle cells [24], platelets [25], and leukocytes [26]. These cells are known to play a role in the development of stroke. Hence, a possible mechanism linking vitamin D levels and stroke occurrence can be accounted for [27]. Animal experiments have found vitamin D to inhibit thrombosis [28], which explains why low vitamin D levels were found to have a higher association with strokes. Moreover, low vitamin D levels are found to up-regulate the renin-angiotensin-aldosterone system (RAAS) [29]. The RAAS is vital for the regulation of the cardiovascular system; its dysfunction may account for the risk of strokes in low vitamin D states [27]. Vitamin D also affects the regulation of various inflammatory mediators like interleukin-6 and tumor necrosis factor-alpha [30]. Its deficiency is considered a poor prognostic indicator in many acute diseases and, hence, can alter the pathophysiology of strokes as well. 

## Conclusions

Ischemic stroke is one of the most dangerous and sudden health problems, affecting a wide range of age groups and both sexes. Hence, it is always considered a medical emergency, and its timely diagnosis requires a strong predictor. Our study showed a significant correlation between vitamin D sufficiency in the body and the incidence of stroke (x2=3.888 and p=0.048), indicating that vitamin D levels can be used as a predictor for the risk of stroke in a person. Also, a significant negative correlation was found between vitamin D level and stroke severity score (NIHSS) at admission (r=-0.2459 and p=0.0335); this proves vitamin D level is an important factor for predicting the severity of stroke in patients. Vitamin D also showed a strong positive correlation with the mRS score at discharge (r=-0.6427, p=< 0.0001), supporting the role of vitamin D in the prognostic outcome of stroke. Other factors, such as diabetes, hypertension, alcoholism, etc., had not shown any significant correlation with the NIHSS and mRS scores. Our study also concluded that the increasing severity of vitamin D deficiency was associated with more deaths and poor outcomes. More studies in this regard can be conducted to predict the role of vitamin D in predicting the outcome of ischemic stroke because the assay is widely available in the majority of hospitals and is inexpensive. Moreover, vitamin D levels may be used as a predictor for the risk of developing ischemic strokes, especially in the developing world, as their testing is low-cost with a quick turnaround time.
